# ICU nurses and physicians dialogue regarding patients clinical status and care options—a focus group study

**DOI:** 10.1080/17482631.2016.1267346

**Published:** 2017-01-05

**Authors:** Monica Kvande, Else Lykkeslet, Sissel Lisa Storli

**Affiliations:** ^a^ Department of Health and Care Sciences, Faculty of Health Sciences, University of Tromsø, The Arctic University of Norway, Tromsø, Norway; ^b^ Faculty of Health and Social Care, Molde University College, Molde, Norway

**Keywords:** Focus group, intensive care nurse, ICU, physician: exchange of patient information

## Abstract

Nurses and physicians work side-by-side in the intensive care unit (ICU). Effective exchanges of patient information are essential to safe patient care in the ICU. Nurses often rate nurse-physician communication lower than physicians and report that it is difficult to speak up, that disagreements are not resolved and that their input is not well received.

Therefore, this study explored nurses’ dialogue with physicians regarding patients’ clinical status and the prerequisites for effective and accurate exchanges of information.

We adopted a qualitative approach, conducting three focus group discussions with five to six nurses and physicians each (14 total).

Two themes emerged. The first theme highlighted nurses’ contributions to dialogues with physicians; nurses’ ongoing observations of patients were essential to patient care discussions. The second theme addressed the prerequisites of accurate and effective dialogue regarding care options, comprising three subthemes: nurses’ ability to speak up and present clinical changes, establishment of shared goal and clinical understanding, and open dialogue and willingness to listen to each other.

Nurses should understand their essential role in conducting ongoing observations of patients and their right to be included in care-related decision-making processes. Physicians should be willing to listen to and include nurses’ clinical observations and concerns.

## Introduction

Nurses and physicians work side-by-side in the intensive care unit (ICU), but they have different roles and sources of knowledge. In the modern approach to critical care, collaboration and patient responsibility are shared among the nurses and physicians in ICU teams (Hartog & Benbenishty, 2015; Søreide & Flaatten, 2010).

Bedside nurses play a fundamental role in ensuring patient safety and preventing patient conditions from declining by conducting ongoing clinical examinations (Livesay, 2016).

The guidelines of the European Society of Intensive Care Medicine (ESICM) state that “Intensive care medicine is the result of close cooperation among physicians, nurses, and allied health professionals (AHCPs)” (Valentin & Ferdinande, 2011). An effective collaboration between nurses and physicians is thus essential for ensuring high-quality health care and patient safety in the ICU (Dietz et al., 2014; Douglas et al., 2013; Hartog & Benbenishty, 2015) and may improve patient outcomes (Martin, Ummenhofer, Manser, & Spirig, 2010). Laerkner, Egerod, and Hansen (2015) investigated nurses’ experiences caring for critically ill, non-sedated, mechanically ventilated ICU patients and found that close collaboration between nurses and physicians was vital to ensuring patient comfort during mechanical ventilation.

Collaboration includes communication, and accurate and effective communication of patient information is an essential component of safe, efficient and patient-centered ICU care (Al-Qadheeb et al., 2013; Williams et al., 2010). Benner, Hooper-Kyriakidis, and Stannard (2011) reported that conveying clear, well-documented patient trends and responses is relatively easy but that excellent communication practices require early unconfirmed recognitions of patient changes to be communicated as well. Furthermore, there is consensus within the ESICM that staff meetings with physicians, nurses and AHCPs must be regularly organized to discuss difficult cases, ethical issues and protocols and to share information (Valentin & Ferdinande, 2011). However, the communication and information exchange that occurs between ICU caregivers is often complicated by the frequent handoffs of patient care, the fluctuating nature of critical illness, the complex therapeutic interventions administered and the highly technical monitoring systems used (Collins, Bakken, Vawdrey, Coiera, & Currie, 2011).

Understanding daily care objectives, which entails discussing patient information, planning patient care and making health-related decisions, is the main foundation of ICU management. In addition, checklists, daily goal sheets, protocols and interdisciplinary rounds are tools that improve inter-professional communication and collaboration in ICUs (Rose, 2011). Recent studies show that physicians and nurses have different perceptions of the quality of nurse-physician collaborations and communication. Specifically, nurses consistently rate the quality of collaboration to be lower than that reported by physicians. Nurses often feel that it is difficult to speak up, that disagreements are not appropriately resolved and that their input is not well received by physicians (Al-Qadheeb et al., 2013; Hartog & Benbenishty, 2015; Nathanson et al., 2011). Through a survey of both nurses and physicians in two medical ICUs, Al-Qadheeb et al. (2013) reported differences in the perceptions of communication between nurses and physicians regarding pain, agitation and delirium that occurred at night and found that bedside nurses often believed that the physicians did not appreciate the urgency or complexity of the particular clinical situation that they had contacted the physician to discuss.

According to Benner et al. (2011), presenting unclear patient changes, patient concerns and early warnings to physicians requires trust, respect, willingness to listen, and the ability to identify subtle changes that indicate transitions in the patient’s condition. Kvande, Delmar, Lykkeslet, and Storli (2015) found that nurses anticipated and became aware of early changes in patients’ clinical conditions through images composed of signs that were sensory and measurable, and manifested as the mood of the nurse. The authors also reported that nurses may have found it difficult to interpret sensory input or to express in words what the change entailed.

There is a need to better understand the verbal communication of ICU nurses when conveying patient information to physicians from both the ICU nurses’ and physicians’ perspectives. Therefore, the aim of the present study was to explore nurses’ dialogue with the physicians on shift regarding patients’ clinical status and the prerequisites for an effective and accurate exchange of information.

## Methodological approach and method

A qualitative design was selected and included focus group discussions with the participants. This study was part of a larger qualitative investigation of the experiences of intensive care nurses that focused on the identification of changes in patient conditions and how these changes were communicated to attending physicians. A field study was conducted that included close observations of bedside nursing practices and in-depth interviews with nurses after their shifts.

One of the main advantages of focus group discussions is that the group interactions that occur can provide insight into a range of opinions, perceptions or feelings that people have about a specific issue, practice or idea that would be less accessible in one-on-one interviews (Doody, Slevin, & Taggart, 2013a; Krueger & Casey, 2014). Furthermore, focus groups can identify the factors that influence opinions, behaviors or motivations in a collective context. Focus groups enable a more natural environment, as participants influence and are influenced by others as they are in real-life settings (Krueger & Casey, 2014). The group dynamics and interactions were expected to help the nurses and physicians clarify their perceptions of nurses’ unique contributions to the exchange of patient information and the factors influencing the communication and reception of this information.

### Setting and participants

We conducted three focus groups with ICU nurses and physicians at two ICUs in two Norwegian university hospitals. Each focus group consisted of both nurses and physicians who worked at the same hospital and included five to six participants (a total of 14). According to Krueger and Casey (2014), the ideal size of a focus group is between five and eight participants, and the accepted standard is to conduct three or four focus group discussions. The inclusion criteria for nurses were having a diploma in intensive care nursing (90 credits) and a minimum of 5 years of practice in an actual ICU, and the inclusion criterion for physicians was rotating on an ICU shift caring for adult patients.

The head of each ICU (nurse and physician) emailed detailed study information and an invitation to participate to selected nurses and physicians in the two ICUs. Those who agreed to participate returned the written consent in a prepaid envelope addressed to one of the researchers (MK).

### Data collection

One moderator and one assistant moderator conducted the three focus groups in the spring of 2013 in a meeting room at the hospital that was convenient for study purposes. The participants were seated around a table to indicate the equal importance of each participant’s contributions. The focus groups lasted between 76 and 83 minutes, which is a common duration of focus group interviews (Krueger & Casey, 2014). The moderator introduced the topic and encouraged the participants to speak freely about their experiences. Emphasis was placed on asking simple, open-ended and clear questions (Krueger & Casey, 2014). The opening question in each of the three focus groups was as follows: “Can you please tell me what patient information you perceive to be essential to share with one another?” This was followed by a discussion of the following themes: nurses’ unique contribution of patient information in the ICU and the prerequisites for the communication and reception of patient information. The same questioning route was used in all of the focus groups.

The moderator directed the discussions to ensure that the appropriate themes were discussed and that all participants were encouraged to contribute to the themes. Examples of the prompts used to obtain additional information and clarify opinions included the following: (Krueger & Casey, 2014) “Could you give an example?”, “Please describe what you mean” and “Tell us more” (p. 120). The participants were actively engaged in the discussions, and their thoughts were sometimes expressed in half sentences that were completed or expanded upon by other participants. The atmosphere was positive, including both humor and laughter.

As recommended by Krueger and Casey (2014), the assistant moderator took field notes during the interviews to capture the themes and key points along with insightful quotes as completely as possible. In addition, the field notes were used to capture non-verbal behavior and to differentiate between speakers and tone within the group.

Near the end of the discussion, the assistant moderator provided a short summary of the key points raised during the discussion and invited the participants to include any additional comments by asking a final, open question: “Is there anything else we should add?” This is an important question, as it can stimulate additional and important discussion points (Doody, Slevin, & Taggart, 2013b).

All of the interviews were audiotaped and transcribed verbatim after each interview by the first author of this manuscript.

### Ethical considerations

This study was approved by the Norwegian Social Science Data Services (NSD) and the participating ICUs. Prior to participation, all participants received verbal and written information regarding the study and the presentation of the results. All of the nurses and physicians provided voluntary written informed consent.

### Data analysis

Krueger and Casey (2014) state that focus group analyzes should consist of four distinct and critical characteristics: they should be systematic, completed in a sequential manner, with verifiable procedures and in a continual process (p. 161). Furthermore, qualitative analyzes do not occur linearly, as one part of the process can overlap another.

The analysis was performed using Doody, Slevin, and Taggart (2013c) concept of analysis, which was based on Kruger and Casey’s (2014) framework. This method comprised six steps: (1) *generating rich data*, in which the moderator skillfully facilitated the discussion and gathered rich data from the discussion; (2) *familiarizing oneself with the data*, in which all researchers read the transcripts and observations of the discussion in their entirety several times to immerse themselves in the data and gain a sense of the text as a whole before dividing it into relevant parts; (3) *writing memos*, in which the researchers wrote short notes in the margins of the text to describe the ideas or concepts that emerged from the text (Doody et al., 2013c; Krueger & Casey, 2014); (4) *indexing*, in which the text was re-read in greater depth and the researchers began organizing the data by highlighting and separating parts of the text that were related to the study aims; (5) *forming themes*, in which the researchers searched for recurring themes in the transcripts and notes that represented patterns and themes across groups. Quotes that were similar in the original text were then rearranged under temporary corresponding themes using the following questions: “Is this text similar to something that has been said earlier?” and “Is it similar to or different from other themes?” (Krueger & Casey, 2014); and (6) *mapping and interpretation*, in which the text was read again and the researchers together reflected on the themes with the goal of interpreting the text as a whole and attaining a comprehensive understanding. In this phase (Doody et al., 2013c), we drew on Gadamer (1975/2004) circle or spiral of understanding, the principle of the whole and the parts, and the interactive process between pre-understanding and understanding. Based on the Gadamerian approach, our pre-understandings were essential for moving from the parts to the whole and from the text to a new understanding.

In the discussion, we relate our findings to relevant research and to Gadamer (1975/2004) work on understanding and on the fusion of horizons.

## Findings

The findings are presented through two themes that reflect the essential aspects related to the study aims. The first theme focused on nurses’ contribution to the dialogue with physicians on shift. The second theme pertained to the prerequisites for an accurate and effective dialogue regarding patients’ clinical status and care options and was further divided into three subthemes: nurses’ ability to speak up and present clinical changes; the establishment of a shared goal and clinical understanding; and open dialogue and willingness to listen to each other.

Our findings are illustrated in the following text by excerpts from the focus group discussions. To facilitate transparency in the reporting of comments and agreement among participants, each participant is referred to by a unique, non-identifying study ID (e.g., nurse 1–8 FG1-3 and physician 1–6 FG1-3).

## Nurses contribution to the dialogue

### Nurses’ ongoing clinical observations—an essential contribution

The participants of the three focus groups agreed that nurses’ ongoing clinical observations of the patient were essential contributions to the exchange of patient information between nurses and physicians. These observations were important in helping the physician develop an impression of the patient’s clinical condition, thereby highlighting the need for nursing input in decision-making processes. The following excerpt demonstrates this idea:

Physician 2 FG1: “I [the physician] feel completely dependent on the nurses’ clinical observations of the patient. I wonder how patients respond to different care situations. How did they respond to oral hygiene? Was there facial mimicry or convulsions? (…) It is important to know how patients’ physical conditions are manifested from hour to hour (…) Nurses conduct ongoing clinical observations of the patients, and those observations are the most important [contribution] …”

Nurse 1 FG1: “I [nurse] continuously follow [up with] the patient … I am aware of the changes in the patient’s skin condition. I look for more facial mimicry and other body movements. How are the patient’s thorax movements? Is the patient low in oxygen saturation? Are there phlegm and mucus sounds that can explain it [low oxygen saturation]? I look at the monitor and the electrocardiogram [ECG]—does the patient have a normal heart rhythm or is there arrhythmia. I look at the ventilator and observe volumes, pressures … I look at the extracorporeal membrane oxygenation [ECMO] flow …”

Physician 5 FG3: “We [physicians] are totally dependent on nurses’ observations. They [ICU nurses] are at the patient’s bedside all the time (…) They observe a lot and document many findings … We are completely dependent on their observations in obtaining an impression of the patient and making a treatment plan…”

Data from bedside monitors and computerized information systems including blood pressure, pulse, intracranial pressure, heart rhythm and other measures provided information about the patient’s clinical conditions and response to interventions. However, the physicians found it difficult to base their assessments solely on these measurable parameters and scales and highlighted the need for nurses’ clinical interpretations of the patient:

Physician 5 FG3: “We can try to quantify the depth of sedation using different scales, but it is not enough just to say −3 or −2. It is not that easy… It [the assessment] is more than just numbers…”

Physician 6 FG3: “In terms of the sedation level of the patient, we [physicians] can hear nurses say, ‘I think that the patient is a little more awake or is reacting in a slightly different way than before’. That [observation] is a little unspecific … It is difficult to put a number on it…”

The participants of the three focus groups agreed that it was essential for non-specific observations or any concerns regarding the patient’s condition to be shared and discussed with each other.

## The prerequisites for an effective and accurate dialogue

### Nurses’ ability to speak up and present clinical changes

The nurses stated that they possessed a substantial amount of knowledge about the patient and highlighted the importance of being able to speak up and present their observations to the attending physicians. They also reported that nurses have to present their opinion and any disagreements to physicians regarding the clinical decisions and actions. The following quotes are from focus group 1:

Nurse 2 FG1: “We [nurses] have a lot of knowledge (…) We have to take a stance and speak up and say ‘listen, there is actually such and such [occurring]’; if there is something we are frustrated about or do not agree with regarding the patient’s care, we must speak up and convey these things to you [physicians]…”

Nurse 2 FG1: “I think I communicate well with the physicians. I do not always get approval for the things I suggest, but I have a lot of experience… I am not afraid to say what I think and to ask questions.”

The nurses stated that it was important to help inexperienced nurses present patient changes, patient concerns and practical issues to the physicians.

Additionally, nurses highlighted the need to ensure that all nurses were aware of the significance of their presence and participation in important discussions about treatment and care options:

Nurse 1 FG1: “I say that very often to new nurses. You have to speak up and be heard [by physicians]. You have to come to the pre-rounds and rounds, as well as interdisciplinary meetings, and present your opinions and what you have observed… Bring them [opinions and observations] up and join the discussion [with physicians]…”

Nurses described sensing that there were changes in the patient’s condition that indicated a negative development but finding it difficult to explain to others what the changes were:

Nurse 7 FG3: “I [nurse] can sense that there is a change in the situation … Sometimes I have no idea what it is … I can sense that there is a difference in the patient’s conditions, that something is not right (…).”

### Establishment of a shared goal and clinical understanding

Both nurses and physicians expressed the need for straightforward, unambiguous verbal exchange of patient information regarding treatment and care options. The nurses highlighted that knowing the treatment plan and the rationale for the interventions helped them gain a clinical understanding and to perform relevant observations of the patient. The following excerpt is from focus group 2:

Nurse 5 FG2: “It is important for you [physician] to communicate the goals of the treatment plan. If the nurse does not know what the intention is [of patient care], it is difficult to have the same objective…”

Physician 4 FG2: “When we [physicians] make conclusions and decide on a medical treatment, it is important for us to communicate why we are doing the things we do. I completely agree with you [nurse 5]; it is very important that this is communicated by us [physicians]. Why we do what we do, why we think what we think—communicating those ideas allows it [treatment plan] to make sense in a way…”

Nurse 5 FG2: “It is one thing to read the orders on a form and to implement them; it is another thing when the physician has explained what their intention is [with the orders]. It is not always easy to know what you [physicians] are asking for…”

### Open dialogue and willingness to listen to each other

Nurses and physicians did not always agree on the next step in patient care, although physicians trusted and valued the nurse’s clinical observations of the patients. Physicians stated that they were the ones who determined whether the nurses’ observations of the patient had clinical significance. In addition, physicians highlighted that they had to assess the patient themselves to obtain their own clinical judgment and to select a patient treatment plan. Nurses, on the other hand, expressed that it was important to be respectful and open to each other and to discuss different opinions regarding patient care. This sentiment was reflected in the following exchange:

Physician 6 FG3: “It is the physicians who determines whether the observations [nurses’ observations] have clinical significance (…) Nurse observations are very important, and we [physicians] trust them, but we must also assess [the patient] ourselves …”

Physician 5 FG3: “I agree … One thing is to present observations of the patient; most nurses have an idea of what they think we [physician and nurses] should do with them [observations], but we [physicians] do not always agree with the nurses… We agree about the observations, but there is a disagreement about what the next steps should be…”

Nurse 7 FG3: “I think the most important thing is that we [physician and nurses] are open, respect different points of view and can discuss various issues. (…)”

The differences in clinical experiences between nurses and physicians made it difficult to agree on actions and treatment strategies. Several of the nurses felt the need to be included in the discussions of decisions about treatment options. The nurses stated that their reflections on the patient’s condition and physician’s willingness to listen to nurse’s clinical perspectives on patient care, such as weaning a patient from a respirator, were important to reaching the best decisions for the patient:

Nurse1 FG1: “It can be difficult … Nurses have suggestions based on their past experiences with similar patient care situations. If the physician on shift does not have the same experience, he may have another suggestion… It can be difficult to agree [with the physicians], and one [nurse] can think that there is another way to do it… I think that there are things we can discuss more often, such as finding a way that is safe to wean a patient from the respirator …”

## Discussion

This study sheds light on ICU nurses’ dialogue with physicians on shift regarding patients’ clinical status and the prerequisites for an effective and accurate exchange of information. Physicians highlighted the value of nurses’ input of ongoing patient observations in helping them establish an impression of the patient’s clinical condition and in making treatment-related decisions. However, our findings showed that the value of nurses’ observations and input depended on whether physicians and nurses shared the same objectives and whether the physicians thought that the nurses’ observations had clinical relevance. Nurses’ ability to speak up and present changes in the patient’s condition was essential to the communication of patient information to physicians. In addition, both nurses and physicians highlighted the need to be mutually open and respectful and to listen to different perspectives when sharing patient information and discussing treatment and care options.

Nurses, who are typically by the patient’s bedside for hours, are able to follow patient situations as they develop, identifying whether patients are declining or are stable and improving. We found that both nurses and physicians perceived nurses’ communication of their clinical observations of the patient to be fundamental to the discussion of medical and nursing care. In other studies (Al-Qadheeb et al., 2013; Hartog & Benbenishty, 2015; Nathanson et al., 2011), nurses often report that their input is not well received and that physicians do not appreciate the urgency or the complexity of the situation that they were trying to communicate. In our study, we found that physicians’ willingness to include and value nursing input in the decision-making process depended on whether they felt that the nurses had focused on the most important aspect of the patient’s condition and reported clinically relevant observations. This may lead to the exclusion of important nursing perspectives from clinical decision-making processes and patient care plans. Regarding this hierarchical relationship, nurses should be aware of their responsibility to speak up, present their clinical observations and interpretations and join discussions on patient care.

Nurses and physicians stated that having experience could make it easier for nurses to share their opinions on a patient’s clinical status and response to treatment and have them be heard by physicians. Nurses reported that increased emphasis had to be placed on enhancing inexperienced nurses’ ability to present patient information to physicians and encouraging them to present opinions and any patient information they consider relevant. This observation is consistent with that of Benner et al. (2011), who stated that reflections on communicating clinical knowledge are needed in high-intensity, high-demand work settings.

Benner et al. (2011) reported that excellent communicative practices require the ability to identify subtle changes that indicate transitions in a patient’s condition and effectively communicating these clinical findings to others. This is similar to the results of other studies (Al-Qadheeb et al., 2013; Williams et al., 2010), which have demonstrated that accurate and effective communication of patient information in the ICU is an essential component of safe and efficient patient care. We found that although nurses may be able to sense changes in a patient’s clinical condition, they may find it difficult to clearly express what those changes are; this perception that something may be wrong with a patient leads to the nurses’ concerns. This observation is similar to that of Kvande et al. (2015), who reported that ICU nurses can sense that a patient’s condition may be changing but may find it difficult to interpret the signs and express them in a way that others would understand.

The physicians in our study stated that for the patient information conveyed by nurses to be effectively received, nurses and physicians had to have the same objectives and understanding of the patient’s clinical status and care options. On the other hand, the nurses stressed that it was important for the physicians to share their medical knowledge and goals for treatment to help them conduct relevant observations of the patient. This finding is similar to the ESICM recommendations, which state that ICUs must organize an efficient communication process between medical and nursing ICU staff and that tasks and responsibilities must be clearly defined (Valentin & Ferdinande, 2011).

As Gadamer (1975/2004) explained, a “fusion of horizons” occurs through a dialogue in which one’s own personal horizons are expanded through a conscious integration of the horizon of the other. With regard to our findings, the corollary is that understanding occurs when nurses and physicians change their current understanding or horizon to a new understanding or horizon because of an encounter. A “fusion of horizons” seemed to illustrate what occurred when nurses and physicians shared different perspectives and patient information and attained a new understanding or horizon. However, there are aspects of the culture of knowledge and work that seemed to influence the dialogue between nurses and physicians. We found that physicians and nurses used different types of knowledge and had different perspectives on the definition of valid knowledge; these discrepancies could lead to a lack of understanding, a lack of communication, or dismissal of patient information. This observation is similar to the findings of Alexanian, Kitto, Rak, and Reeves (2015), who demonstrated that different cultures, hierarchies within and between professions, and medical dominance influenced how work occurred in the ICU. Hartog and Benbenishty (2015) also reported similar findings, stating that true collaboration required all disciplines within the team to be considered equal partners with different roles and knowledge. Al-Qadheeb et al. (2013) found that ICU nurses and physicians often assigned a different level of urgency to the same clinical situation of pain, agitation, or delirium. We found that nurses and physicians did not always agree on the next step in patient care, although the physicians trusted and agreed with the nurses’ clinical observations of the patient. Nurses expressed the importance of having mutual respect and an openness to asking questions and discussing different perspectives on the patient’s care. According to Gadamer (1975/2004), understanding begins when something “addresses” us. This event requires the suspension of our own prejudices, which then translates into the logical construction of a question. The purpose of the question is to open possibilities and keep them open (p. 310). In terms of our findings, this suggests that effective nurse-physician communication requires an openness and willingness on the part of physicians to value and include nurses’ clinical observations and patient concerns in the discussion of patient care. This observation is similar to the findings of Benner et al. (2011), who highlights that communicating unconfirmed judgments and early warnings requires trust, respect and willingness to listen. As Gadamer (1975/2004) explains, the development of a new understanding occurs through a dialogue of questions and answers in which we fully participate, conscious of our own preconceptions and history. With regards to our findings, this implies that if there is openness between nurses and physicians in the decisions made for the patient as well as a willingness to respect and include the other’s input, nurses’ and physicians’ information about a patient can complement each other and lead to a shared understanding of the treatment plan and care options.

## Strengths and shortcomings

One limitation of the present study could be its small sample size. However, our aim was not to generalize the findings. Another limitation of the present study could be the inclusion of participants who already knew each other. However, by using pre-existing groups, one is able to observe some aspects of their interactions, and this can approximate naturally occurring data, such as data collected through participant observation. An additional advantage of this approach is that nurses and physicians were able to relate each other’s comments to actual incidents in their shared daily work lives. They could challenge each other on contradictions between what they said they believe and how they actually behave (Krueger & Casey, 2014).

The inclusion criteria for nurses and physicians regarding work experience differed, and these differences in clinical experience could have affected the discussion. However, the nurses and physicians included in the study all had a minimum of 5 years of practice in an actual ICU.

We acknowledge that ICU settings and work routines vary by country and throughout different parts of the world. Nurse and physician staffing models, nursing education, and decision-making hierarchies (Rose, Dainty, Jordan, & Blackwood, 2014) may all differ, potentially limiting the transferability of our findings; however, this study illustrates an example of what may be occurring in clinical ICU practice.

The preconceptions of the moderator and assistant moderator influenced both the questions raised and the analysis. However, we aimed to maintain a balance between remaining close to the themes in the data, as an essential component of the generation of understanding, and striving for sensitivity about unavoidable preconceptions, which involved reflexivity.

## Conclusion and implications for practice

This study offers insight into ICU nurses’ dialogue with physicians on shift regarding patients’ clinical status and the prerequisites for an effective and accurate exchange of information.

Nurses should be aware of their essential role in conducting ongoing clinical observations of patients and their right to be included in decision-making processes regarding patient treatment and care. We believe that this study underscores the need to strengthen nurses’ ability to report their clinical observations and interpretations to the physicians on shift. This requires an increased emphasis in the education system and in nursing practice on how to present potential patient changes and patient concerns. In addition, accurate and effective dialogue between nurses and physicians on shift requires leadership that is able to organize routine interdisciplinary meetings. Furthermore, this necessitates a willingness on the part of physicians to listen to and include nurses’ clinical observations and concerns about a patient in the decision-making process.
